# Silicon-based double fano resonances photonic integrated gas sensor

**DOI:** 10.1038/s41598-024-74288-6

**Published:** 2024-10-22

**Authors:** Norhan A. Salama, Shaimaa M. Alexeree, Salah S. A. Obayya, Mohamed A. Swillam

**Affiliations:** 1 Laser Applications in Metrology, Photochemistry and Agriculture, National Institute of Laser Enhanced Sciences, Cairo University, Giza, Egypt; 2 Department of Physics, School of Science and Engineering, The American University in Cairo, Cairo, 11835 Egypt; 3 Centre for Photonics and Smart Materials, Zewail City of Science, Technology and Innovation, Giza, Egypt

**Keywords:** Metasurfaces, Metamaterial, Double Fano resonance, CO gas sensor, N_2_O gas sensor, Optical gas sensor, Engineering, Materials science, Nanoscience and technology, Optics and photonics, Physics

## Abstract

The telecommunication wavelengths are crucial for developing a photonic integrated circuit (PIC). The absorption fingerprints of many gases lie within these spectral ranges, offering the potential to create a miniaturized gas sensor for PIC. This work presents novel double Fano resonances within the telecommunication band, based on silicon metasurfaces for selective gas sensing applications. Our proposed design comprises periodically coupled nanodisk and nanobar resonators mounted on a quartz substrate. Fano resonances can be engineered across the range from λ = 1.52 μm to λ = 1.7 μm by adjusting various geometrical parameters. A double detection sensor of carbon monoxide (CO) at λ = 1.566 μm and nitrous oxide (N_2_O) at λ = 1.674 μm is developed. The sensor exhibits exceptional refractometric sensitivity to CO of 1,735 nm/RIU with an outstanding FOM of 11,570 at the first Fano resonance (FR1). In addition, the sensor shows a sensitivity to N_2_O of 194 nm/RIU accompanied by an FOM of 510 at the second Fano resonance (FR2). The structure reveals absorption losses of 6.3% for CO at the FR1, indicating the sensor selectivity to CO. The sensor is less selective at FR2 and limited to spectral shifts induced by each gas type. Our proposed design holds significant promise for the development of a highly sensitive double-sensing refractometric photonic integrated gas sensor.

**Supplementary Information:**

The online version contains supplementary material available at 10.1038/s41598-024-74288-6.

## Introduction

The design of smart, compact, and low-cost gas sensors is a growing demand in modern society, as they play a crucial role in environmental monitoring^1^, industrial safety^2^, food safety^3^, disease diagnosis^4^, and medical applications^5^. In particular, sensing carbon monoxide (CO) is of pivotal importance as a serious pollutant greenhouse gas. CO is a colorless and odorless gas that introduces significant harmful effects on human health^6,7^. CO is generated as a byproduct of the incomplete combustion of fusil fuel and organic material in industrial processes, transportation, and residential applications. Other kinds of gases are useful for medical applications, such as nitrous oxide (N_2_O). N_2_O is used as an anesthetic in dental surgery and ambulances. However, the overdose of N_2_O causes dissociative anesthesia and a lack of oxygen levels in the body^5^. In addition, CO and N_2_O have an adverse impact on climate change and ozone layer degradation^8^.

Various techniques are employed for gas sensing applications, such as the quantum cascade laser spectrometer^9^, charge transfer effect^10^, and the electrical resistance changes of metal oxide-based sensors^11^. Despite the advantages of high sensitivity and cost-effectiveness, these methods suffer from low chemical specificity, poor scalability, and limited longevity.

On-chip optical gas sensors offer an alternative approach to tackle the aforementioned limitations. These sensors are based on enhancing light-matter interaction through the creation of confined hotspots of evanescent fields of nano/ micro-structures^12,13^. Two main optical sensor platforms exist, namely the refractive index (RI), with high sensitivity portability, and low cost, but at the expense of selectivity, and the absorption sensor, with high sensitivity and selectivity, but by size and cost. Swillam et al.^14^ have demonstrated the possibility of detecting the dispersion of both the real and imaginary parts of the targeted substance using the RI sensors using micro‑ring resonator (MRR) multiple resonances. The dispersion of the complex refractive index is a unique feature of each substance, that can be extracted from the shift of resonance and energy losses.

Near and MID-IR spectral regions are of pivotal importance for sensing applications, as most molecules have unique fingerprints within these ranges^15^. The telecommunication wavelength range in near IR is the best choice for PIC applications. Silicon (Si) photonics offer exceptional performance, such as high-speed data transmission, miniaturization, high sensitivity, and scalability^16^. Moreover, the advancement in fabrication technologies for nano/microstructures and the complementary metal oxide semiconductor (CMOS) process position Si as the best candidate for sensing applications^17^.

To date, the realization of optical gas sensors is mainly based on plasmonic platforms such as Au-CuO nanocomposites and Au-YSZ for CO sensing, though they suffer from significant dissipation losses^18–21^. Despite the undisputed advantages of highly sensitive small designs of plasmonic sensors, the severe inherent dissipation losses engendered by using noble metals act as major obstacles. This is apart from CMOS incompatibility and the material cost^22^.

Thanks to the optical metasurfaces, particularly dielectric metasurfaces, the dissipation losses have been greatly enhanced demonstrating exquisite sensitivity and quality factor accompanied with the low cost and the ease of fabrication^23–27^. Metasurfaces are structures consisting of subwavelength 2D nanoantennas that can be adequately designed introducing phase discontinuity across the surface^28^. For the dielectric metasurfaces, the underline physics is associated with the first and second Mie scattering resonances of the subwavelength resonators. The dielectric resonators demonstrate a strong response to both the electric and magnetic fields allowing full phase coverage from 0 to 2π^22^.

## Sensor performance metrics

For sensing applications, several spectral features should be considered^29^:

First: The sharpness of the resonance wavelength, is expressed by the quality factor (Q-factor). The Q-factor expresses how long the field is confined and interacts more with the analyte. The high Q-factor reduces the spectral noise and hence, enhances the limit of detection (LOD). The Q-factor is defined as:

1 $$Q = \frac{{\lambda_{res} }}{FWHM}$$

where ${\lambda }_{res}$ is the resonance wavelength and (FWHM) is the full width at half maximum.

Second: Sensitivity (S), which is the spectral shift ($\Delta \lambda$) introduced by a change of the refractive index of the surrounding medium $(\Delta n)$, calculated as follows:

2 $$S = \frac{\Delta \lambda }{{\Delta n}} = \frac{{\lambda_{0} }}{{n_{r} }} F$$

where F and ${n}_{r}$ represent the fraction of optical energy of the resonant mode in the analyte and the analyte real part of the refractive index.

Third: LOD represents the minimum detectable change in the refractive index and is calculated as follows^30^:

3 $$LOD = \Delta n_{min} = \frac{1}{{Q S^{{\prime }} }}$$

where $S^{{\prime }}$ is the normalized sensitivity to the resonant wavelength.

Fourth: The figures of merits (FOM) associated with the total Q-factor. The FOM is defined as the ratio between the sensitivity and FWHM of the Fano resonance. It can be expressed in terms of Q-factor and sensitivity to elaborate the tradeoffs between the sensitivity and Q-factor as follows:

4 $$FOM = \frac{S}{FWHM} = Q_{tot} S/\lambda_{0}$$

where ${Q}_{tot}$ represents the total Q-factor of the sensor after exposure to the analyte, which can be related as follows:

5 $$\frac{1}{{Q_{tot} }} = \frac{1}{{Q_{s} }} + \frac{1}{{Q_{a} }}$$

where ${Q}_{s}$ and ${Q}_{a}$ are the Q-factors of the sensor and the analyte, respectively. For an ideal lossless sensor (i.e. ${Q}_{s}$ approaches to infinity), the ${Q}_{tot}$ is defined by ${Q}_{a}$. ${Q}_{a}$ is expressed by the following relation:

6 $$Q_{a} = \frac{{n_{r} }}{{2Fn_{i} }}$$

where ${n}_{r}$ and ${n}_{i}$ are the real and the imaginary parts of the refractive index of the analyte. By subsitiuting Eqs. (2) and (6) into Eq. (4), we can derive the upper limit of the FOM as:

7 $$FOM_{max} = \frac{1}{{2n_{i} }}$$

Equations (4) to (7) indicate that FOM and ${Q}_{tot}$ are decreased by increasing the imaginary part of the analyte. Furthermore, Eq. (4) indicates that the ${Q}_{tot}$ and sensitivity are in trade-offs.

Fifth: The absorption losses (L) after the exposure to the analyte, calculated as follows;

8 $$L = \frac{{FWHM_{a} - FWHM_{0} }}{{FWHM_{0} }}$$

where ${FWHM}_{a}$ and ${FWHM}_{0}$ are the full width at half maximum for the analyte and free space, respectively.

## Optical sensor platforms based on silicon photonics

Silicon photonics offer excellent sensing performance based on various platforms such as silicon photonics microring resonators (RR) as outlined by Chrostowski et al. in ref^30^. As previously mentioned, sensitivity and Q-factor are trade-offs and inversely proportional. Minimizing the intrinsic losses of the sensors relative to the absorption losses of the analyte is crucial for achieving high sensitivity while maintaining a good Q-factor**.** By minimizing intrinsic losses, a smaller change in refractive index due to the analyte can cause a larger shift in the resonant wavelength, leading to higher sensitivity. Platforms based on silicon photonics microring resonators, such as ring/racetrack resonators, slot waveguide resonators, and disk resonators, offer excellent sensing performance with an LOD down to $2.49\times {10}^{-4}$ and a high Q-factors (${10}^{4}$ to ${10}^{5}$) with limited sensitivities (27 nm/RIU to 460 nm/RIU), according to theoretical models. However, Chrostowski et al. have reported that the experimental realization of these sensors imposes further intrinsic losses, such as waveguide scattering loss, waveguide material absorption, and resonator radiation loss. Additionally, side-wall roughness scattering in the TE-like mode devices results in increased losses, reducing the Q-factor. Similarly, a 1D Photonic crystal (PC) platform in TE mode, consisting of an array of 1D drilled hole resonators evanescently coupled to a single bus waveguide, shows an LOD of $7\times {10}^{-5}$, a Q-factor of approximately $3000$, and a sensitivity of 130 nm/RIU. However, this configuration provides field overlap with side-wall roughness, introducing scattering losses. Metasurfaces offer an enhancement in intrinsic losses upon fabrication, although they are limited by the surface states created during etching processes^31^. Furthermore, the metasurface Q-factor is affected by the array size, as the array perturbation at the edge breaks the coherence, leading to strong scattering in free space and broadening the spectrum. For instance, a metasurface consisting of a periodic lattice of rectangular bar and ring resonator demonstrates a sensitivity of 289 nm/RIU and an increased Q-factor as the array size increases, reaching saturation (Q-factor = 489) for an array size of 90,000 unit cells. The simplicity of metasurface designs, less etching in the fabrication process, and less side wall roughness put metasurfaces at the forefront platforms for various photonics applications.

## Fano resonance sensing approach

Fano resonance is one of the most intriguing phenomena that is widely exploited for sensing applications^32,33^. Fano spectral line is characterized by the presence of a dip and peak of the transmission or reflection spectrum showing a high-quality factor^34,35^. Fano resonance results from the coupling of two oscillators with different damping rates. At resonance, the undamped oscillator shows an abrupt π phase shift, while the strongly damped oscillator shows a slow phase change introducing a broad spectral line. Fano spectral resonance has been realized in various configurations such as photonic crystals^24,36^, microcavities^37^, dielectric cylinders^38^, dielectric spheres^39^, and metasurfaces^33^. The periodic configurations such as the photonic crystals and metasurfaces demonstrate narrower spectral lines compared to the single resonators. This phenomenon positioned metasurface, as an easy fabrication material, at the forefront of the sensing applications^34^.

Numerous structure designs have been investigated for gas sensing applications based on the Fano resonance perspective. For example, a side-coupled upright rectangular cavity with a metal-dielectric-metal (MDM) waveguide has been investigated for CH_4_ and H_2_ sensing applications. The structure demonstrates a plasmonic Fano resonance with sensitivity up to 846 nm/RIU and a Q-factor of 1.7^40^. Further study, plasmonic microcavities have been proposed utilizing the doped silicon as a new approach to induce a plasmonic effect with mitigated plasmonic losses. The structure shows sensitivity up to 6000 nm/RIU and FOM of 385 providing limited insertion losses^37^. The same approach is used for aluminum-doped zinc oxide (AZO) metasurface that are used for H_2_ gas sensing showing a redshift ~ 13 nm within 10 min for H_2_ concentration 4%^41^.

On the other hand, structures based on all-dielectric high index material such as the periodic “Lucky knot” shaped nanostructure^42^, split bar resonator^43^, and periodic unit cells of coupled rectangular bar and ring resonators^31^, coupled nanobar with nanodisk^44^, coupled nanoellipse with nano-bar^45^, are employed for sensing applications for different materials. All-dielectric structures show enhanced quality factors reaching up to 980 in some cases^43^, however with less sensitivity than their plasmonic counterparts.

In this work, we present a double Fano resonant metasurface with design-flexible characteristics, enabling easy tailoring of its resonant properties for various gas sensing applications. Our design is based on all-dielectric silicon operating around the telecommunication wavelength (λ = 1.55 μm) for selective gas sensing applications in PIC. The proposed design comprises periodic cells of coupled silicon nanodisk and silicon nanobar resonators. Our work is categorized into five sections; initially, we define the structure geometry and the simulation setup. Secondly, we study the double Fano resonance mechanism showing the near-field coupling effect between the bright mode of the nanobar resonator and the dark mode of the nanodisk/nanobar resonator. The generated Fano resonances are derived from the destructive and constructive interference between the bright mode of the nanobar and the dark mode of either the nanodisk or the nanobar. Thirdly, we study the different geometrical parameters effect, the radius of the nanodisk (r), the gap distance (G), and the nanbar width (w), on the double Fano resonance, covering the spectral range from (λ = 1.52 μm) to (λ = 1.7 μm). The quality factor (Q-factor) of each sweeping parameter is calculated reaching 15,712 for (r = 205nm, G = 180 nm, w = 340 nm). Finally, the sensor is optimized with the geometrical parameters of (r = 205 nm, G = 180 nm, w = 333 nm) for selective sensing of both CO (FR1 at λ = 1.566 μm), and N_2_O (FR2 at λ = 1.674 μm). The sensor achieves an outstanding sensitivity of 1,736 nm/RIU for CO detection accompanied by exceptional FOM of 11,570 and exhibits significant losses of 6.3% following exposure to CO gas. In addition, the sensor exhibits a sensitivity to N_2_O of 194 nm/RIU accompanied by an FOM of 510 and an absorption loss of 2.6% following exposure to N_2_O. The sensor exhibits limited selectivity at FR2, owing to the low Q-factor. Our design fabrication method has been demonstrated in ref^46^. Fabrication is started with the deposition of a 220 nm thick silicon layer on a quartz substrate using low-pressure chemical vapor deposition (LPCVD). Then, the structure is defined using electron beam lithography (EBL). A 10 nm thick chromium (Cr) layer was deposited on top of a polymethyl methacrylate (PMMA) resist layer to serve as a charge dissipation layer. Subsequently, a JEOL 9300FS 100kV EBL tool was used to define the metasurface pattern on the resist. A fluorine-based inductively coupled plasma (ICP) reactive ion etching (RIE) recipe utilizing C_4_F_8_, SF_6_, O_2_, and Ar gas flows was employed to etch the Si layer, transferring the designed pattern onto the underlying substrate.

Owing to the challenge of the required long optical path length for light-gas interaction, several approaches are proposed for achieving miniaturized gas cells with long optical path lengths. Among them, is the impressive approach of using a linear-variable optical filter (LVOF) as a gas cell^47^. The (LVOF) is composed of two face-to-face Bragg mirrors; a flat mirror and a tapered mirror. The (LVOF) acts as an array of Fabry-Pero cavities allowing multiple reflections and hence, increasing the optical path length. Accordingly, we find a strong potential for integration of our design with the (LVOF) allowing a miniaturized device with on-chip scale level.

Our reported design demonstrates superior performance for gas sensing applications compared to the previous studies presented in Table 1.

**Table 1:** Comparison between our sensor and the previously reported Fano resonance sensors.

Structure	Working wavelength	sensitivity	Sensing material	Q-factor	FOM
Coupled plasmonic Si microcavities^37^	3.6 μm	2,300 nm/RIU	(CH_2_O)		60
	4.46 μm	3,860 nm/RIU	(N_2_O)	385	145
Coupled ring/nanobar^31^	1.35 μm	289 nm/RIU	n = 1.4 to n = 1.44	483	103
metal-dielectric-metal (MDM) waveguide^40^	0.948 μm	846 nm/RIU	CH_4_ and H_2_	28	73
Periodic “Lucky knot”^42^	7.3 μm	986 nm/RIU	Glucose at different temperature	520	33
Split bar resonator^43^	1.6 μm to 2μm	525 nm/RIU	n = 1.3 to n = 1.7	800	260
The reported design	1.566 μm	1,735 nm/RIU	CO	15,640	11,570
		194 nm/RIU	N_2_O	4,293	510

## Design and simulation setup

**Fig. 1:**
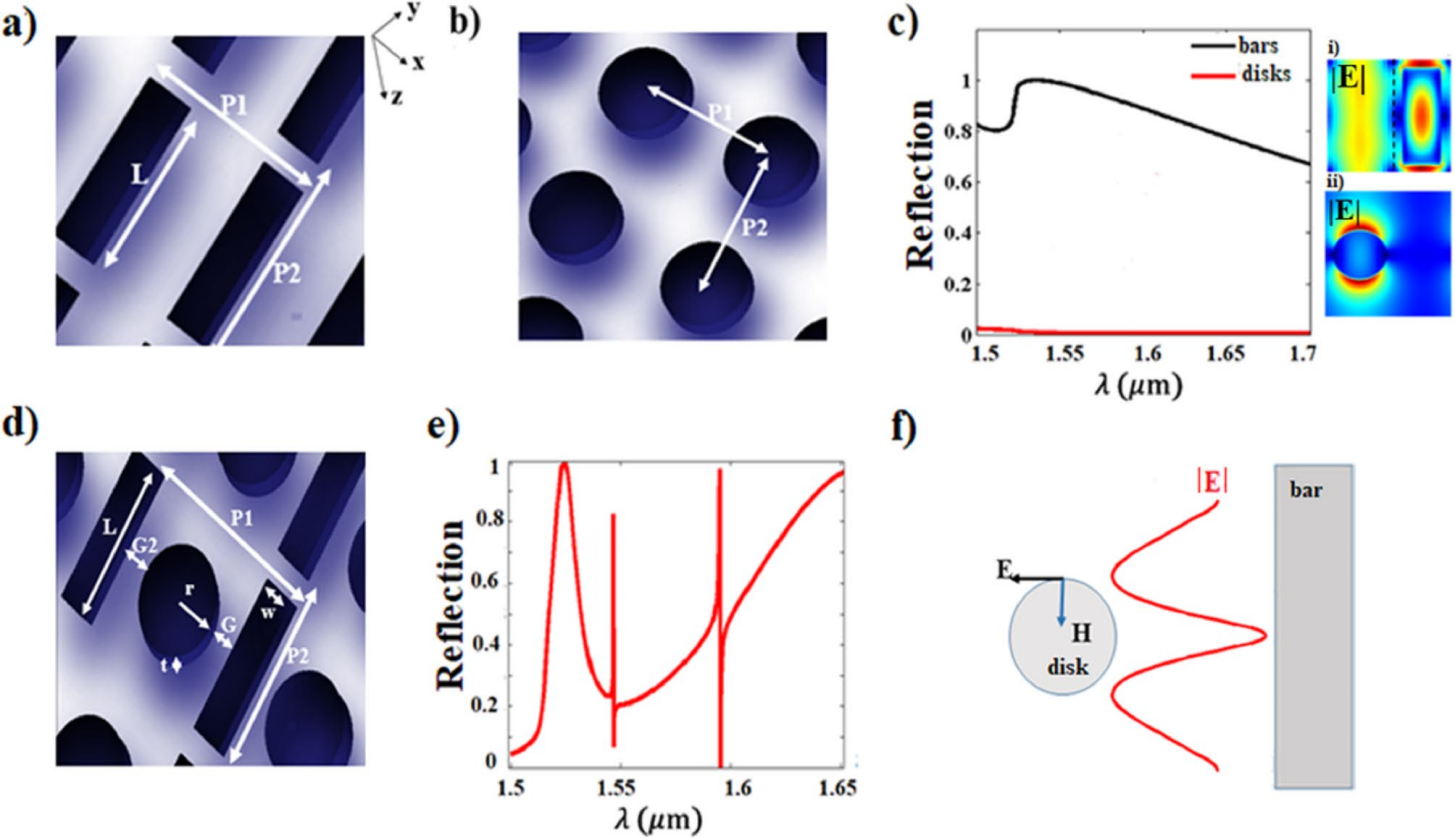
Schematic and normalized reflection spectra of the nanobars, the nanodisks, and the coupled resonators with the dimensions of; r = 205 nm, L = 900 nm, w = 300 nm, G = 130 nm, G2 = 150 nm, P1 = 990 nm, P2 = 1050 nm and t = 220 nm. (**a**, **b**) Schematics of periodic nanobars and nanodisks, respectively. (**c**) The normalized reflection spectrum of both the nanobars (black curve) and the nanodisks (red curve) and their corresponding 2D electric field profiles: (i) the nanobars (the dashed line: Bessel beam-like mode plane), and (ii) the nanodisks. (**d**) Schematic of the coupled nanobars and nanodisks. (**e**) The normalized reflection spectrum of the coupled resonators showing the generated double Fano resonances. (**f**) Diagram showing the near-field dipole coupling mechanism. The generated electric field (red curve) near the nanobar excites the magnetic field inside the nanodisk, which induces a circulating electric field on the nanodisk edge.

Initially, the schematic of periodic nanobars and periodic nanodisks with the corresponding normalized reflection spectra are presented in Fig. 1a–c. The design under consideration is based on periodic coupled oscillators of nanobars and circular nanodisks as depicted in Fig. 1d. The initial geometrical parameters of the structure are defined as; the nanobar length of (L = 900 nm), the width of (w = 300 nm), radius of (r = 205 nm), the gap distance between the resonators of (G = 130 nm), the other side gap distance of (G2 = 150 nm), the pitch in x-direction of (P1 = 990 nm), the pitch in y-direction of (P2 = 1050 nm) and the thickness of both resonators of (t = 220 nm). The structure is mounted on a quartz substrate. The optical response of each system is numerically investigated using commercial software (Lumerical) based on the finite difference time domain method (FDTD)^48^. The simulation setup is established as follows; the periodic boundary conditions are used for x and y directions, while the perfectly matched layers (PML) are used in z-direction. The structure is impinged on by a normal incident plane wave with a polarization direction parallel to the long axis of the nanobar resonator (y-polarization). Silicon refractive index is extracted from Lumerical material library of silicon-Palik and quartz substrate refractive index is extracted from ref^49^. The simulation employs an auto-nonuniform mesh type, configured to a mesh accuracy level of 7, which defines the mesh grid density of 30 points per the effective wavelength. The effective wavelength is inversely proportional to the refractive index, implying a smaller mesh size for high-index material. This mesh accuracy allows for capturing detailed physical phenomena effectively. The simulation setup is validated by the results presented in ref^46^, which includes both simulation and experimental validation, showing excellent agreement, as can be shown in Fig. S1 in the supplementary material.

## The double Fano resonance mechanism

Fano resonance originates from the interference between a continuum band of states with the discrete quantum optical states of coupled two resonators. The coupled resonators response could be treated as a coupled harmonic oscillators model described by the following Eqs. ^31^:

9 $$\begin{gathered} \dot{x}_{1} - j\left( {\omega_{0} + j\gamma_{1} } \right) x_{1} + j\kappa x_{2} = gE_{0} e^{j\omega t} \hfill \\ \dot{x}_{2} - j\left( {\omega_{0} + \delta + j\gamma_{2} } \right) x_{2} + j\kappa x_{1} = 0 \hfill \\ \end{gathered}$$

where ${x}_{1}$ and ${x}_{2}$ are the amplitudes of the collective modes of resonator 1 (bright mode) and resonator 2 (dark mode), respectively. ${\omega }_{0}$ is the central resonance frequency of the bright mode resonator. ${\gamma }_{1}$ and ${\gamma }_{2}$ are the damping rates of the two resonators expressing the radiative and the nonradiative damping. $\kappa$ and $\delta$ are the coupling coefficients between the resonators and the detuning of resonance frequency of oscillators 1 and 2, respectively. $g$ is the dipole coupling strength of the bright mode with the incident electric field ${E}_{0}$. The interaction between the bright resonance with the narrower dark resonance, which coexists at a certain spectral range, leads to high-quality factor Fano spectral line.

Damping rates and the coupling coefficient are crucial precursors for the Fano resonance phenomenon. In our design, the nonradiative damping rates are suppressed due to utilizing an all-dielectric material. Simultaneously, the radiative damping is minimized due to the collective oscillation of the array unit cells. On the other hand, decreasing the coupling coefficient $\kappa$ than the larger damping rate, which is dependent on the geometrical parameters, is necessary to enhance field localization and consequently increase the Q-factor. The interplay between damping rates and coupling coefficient plays a pivotal role in shaping Fano resonances and their corresponding Q-factors in metamaterials.

Silicon-based circular nanodisks have been previously explored providing the possibility of introducing a Fano resonance that is controlled by the aspect ratio; the diameter and the height of the disk, enabling high directionality control^38^. The resulting resonance represents the strong coupling between the Mie-like mode and the Fabry-Pero-like mode. However, a dark mode is realized when the radiation from all the modes compensates each other^35^. The nanodisk of the thickness of (t = 220 nm), the radius of (r = 205 nm), and the periodic distance of (P = 990 nm) exhibit dark mode along the range of wavelengths from 1.5–1.7μm as can be observed in Fig. 1c (red curve), consistent with^38^. Building upon the findings in^50^, the dark mode of the nanodisk can be excited by the azimuthal incidence of the near field Bessel beam generating so-called pseudo modes, the modes that have no real optical oscillations. Axially symmetric Bessel beam offers the advantage of exciting specific polarization only i.e. TM or TE mode, eliminating the potential of mode suppression caused by interference.

Different theoretical approaches have been proposed for launching near-field Bessel beams such as the parallel plates waveguide^51^ and metamaterial lens with gradient index^52^. In our design, we utilize a nanobar to generate a Bessel-like mode, as visually evidenced in the 2D electric field profile of the nanobar along the dashed line near the nanobar surface (Fig. 1c, i). The nanobar acts as a bright mode with a wide bandwidth as shown in Fig. 1c (black curve).

When the two resonators are brought in close proximity, as shown in Fig. 1d, double Fano resonances are realized as demonstrated in Fig. 1e. The schematic diagram presented in Fig. 1f is an initial demonstration of the coupling mechanism between the two resonators. The dipole mode of the nanobar (red curve)- the electric field intensity profile along the dashed line in (Fig. 1c, i)- excites the magnetic dark mode within the nanodisk, inducing a displaced perpendicular electric field circulating the nanodisk. A detailed investigation of this coupling mechanism and its role in both Fano resonances (FR1 and FR2) will be presented in the following subsections.

### Fano resonance at FR1

**Fig. 2:**
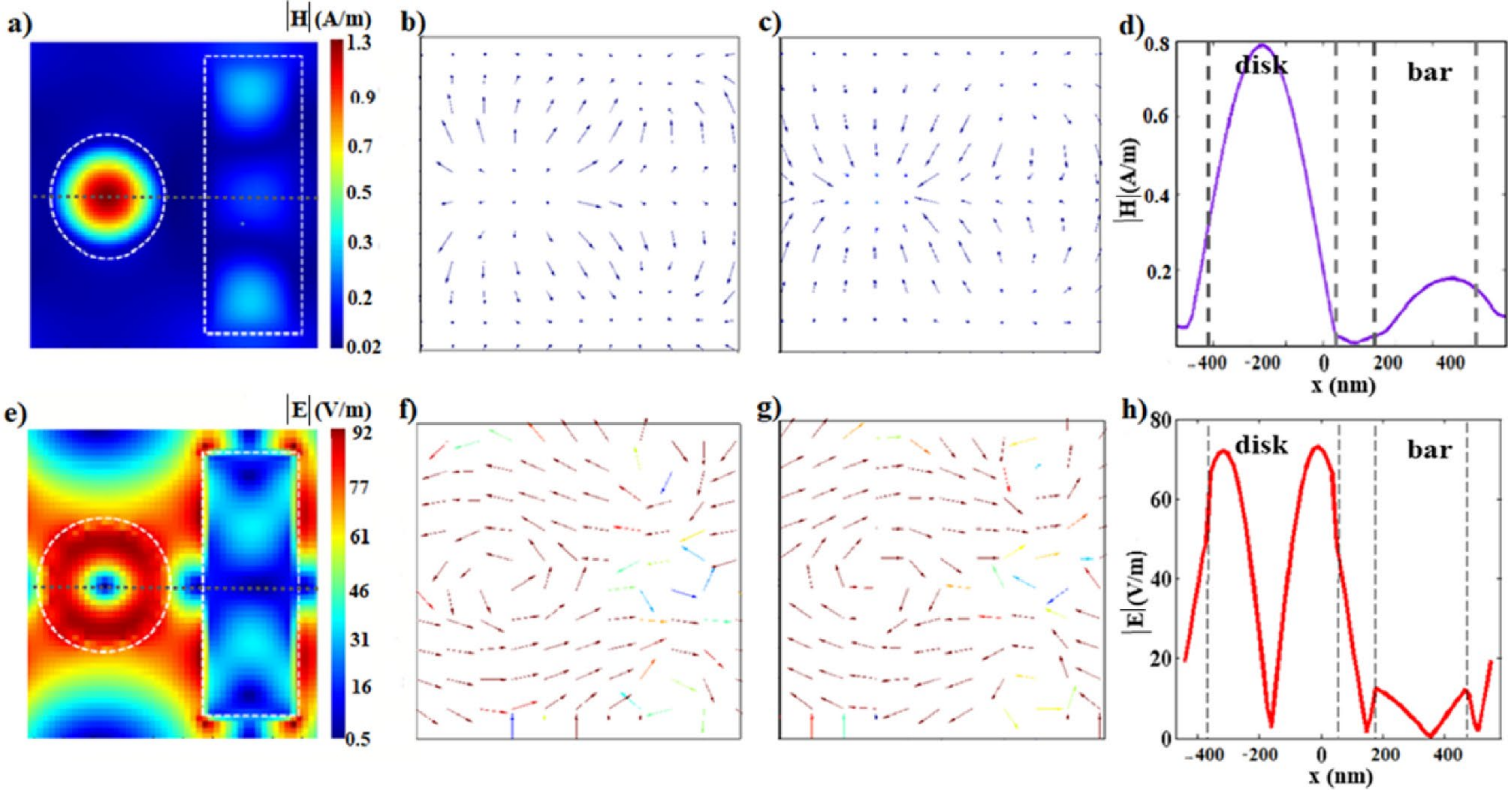
The different H-and E-field profiles at FR1; (**a**) the 2D cross-section of the H-field profile at the peak (λ = 1.548 μm). (**b**, **c**) The vector H-field profiles reveal the field direction at the peak (out-of-plane, λ = 1.548 μm) and at the dip (in-plane, λ = 1.5483 μm). (**d**) the line H-field profile along the horizontal axial symmetry (the gray dashed line in Fig.a). (**e**) the 2D cross-section of the E-field profile. (**f**, **g**) The vector E-field profiles illustrate field circulation around the nanodisk, with opposite directions for the peak and the dip wavelengths. (**h**) the line E-field profile along the horizontal axial symmetry plane (the gray dashed line in Fig.e).

At FR1, the demonstration of the near field coupling mechanism of the nanobar and the nanodisk, explained above, is verified showing a magnetic dipole resonance within the nanodisk, Fig. 2a, inducing a displaced circulating electric field around the nanodisk, Fig. 2e–g. The magnetic and the electric field profiles are characterized by field distribution following the Bessel function, showing a maximum magnetic field intensity at the center accompanied by a minimum electric field intensity at the same position, as depicted in Fig. 2d and h.

In the gap distances between the resonators, the electric and magnetic fields are almost equal to zero. The overall electric and magnetic field distribution demonstrates a spatial Fano resonance. Our numerical study aligns with the analytical solution for the near-field Bessel beam excitation of spherical nanoparticles presented in ref^50,53^. From Fig. 2b–c and f–g, distinct magnetic and electric phase shifts are observed in the frequency domain between the peak (λ = 1.548 μm) and dip (λ = 1.5483 μm) resonances.The magnetic field exhibits an abrupt change in orientation, transitioning from outward for the peak resonance to inward for the dip resonance. Similarly, the electric field circulates in opposite directions for the peak and dip resonances, as shown in Fig. 2f and g.

### Fano resonance at FR2

**Fig. 3:**
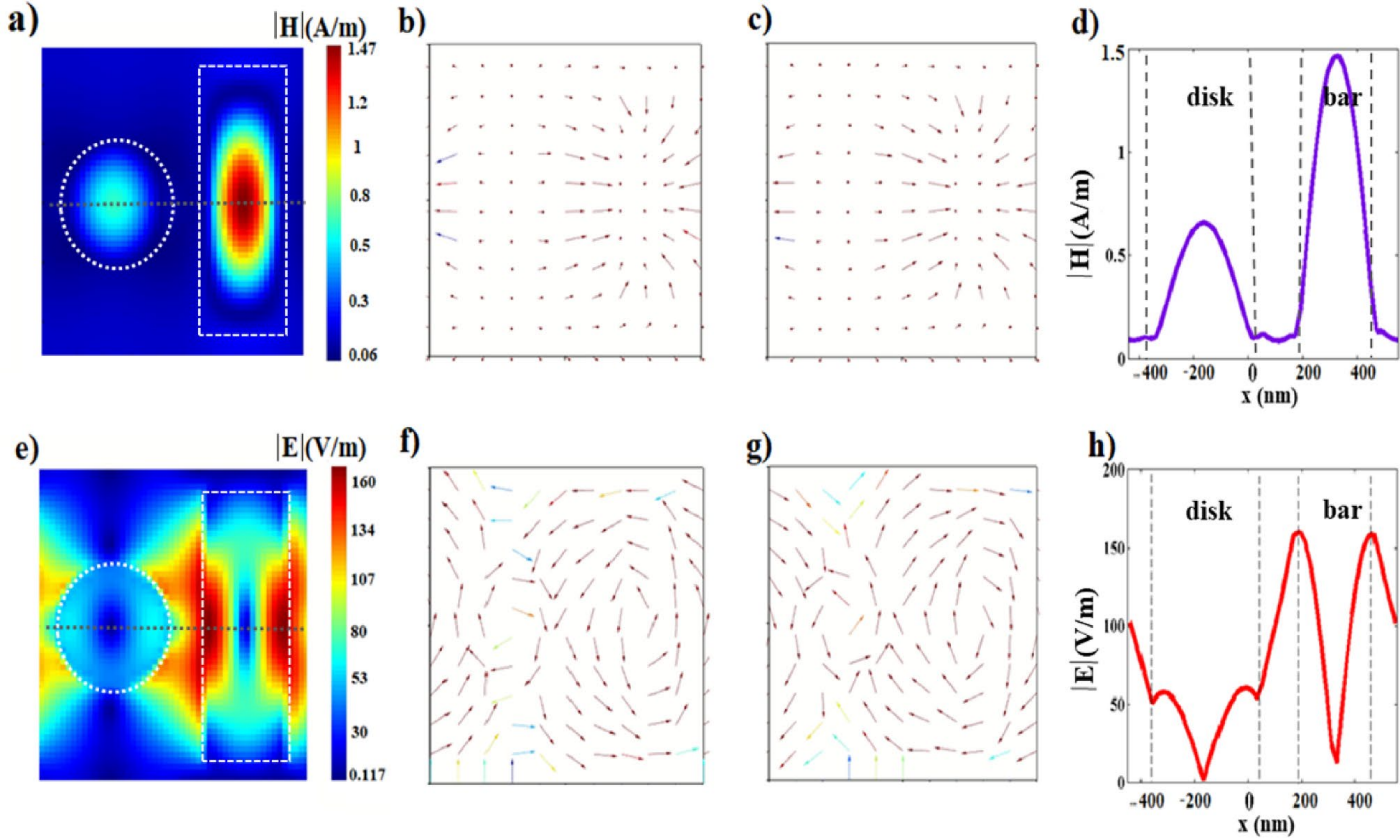
The different H- and E-field profiles at FR2; (**a**) the 2D cross-section of the H-field profile at the peak wavelength. (**b**, **c**) The vector H-field profiles reveal the field direction at the peak and dip wavelengths, respectively. (**d**) the line H-field profile along the horizontal axial symmetry (the gray dashed line in Fig.a). (**e**) the 2D cross-section of the E-field profile at the peak wavelength. (**f**, **g**) The vector E- E-field profiles illustrate the circulating field on both the nanobar and nanodisk. The circulation flips between the peak and dip wavelengths. (**h**) the line E-field profile along the horizontal axial symmetry (the gray dashed line in Fig.e).

At FR2, the nanobar bright mode excites the magnetic dark mode within the nanodisk as well as the magnetic dark mode within the nanobar with different strengths, Fig. 3a and d. Similar to FR1, the out-of-plane magnetic resonance within the nanodisk, Fig. 3b and c, results in a displaced circulating electric field around the nanodisk, Fig. 3f and g. In addition, a strong electric field confinement is generated between the nanobar and the nanodisk and on the edge of the nanobar facing the nanodisk, Fig. 3e and h. The electric field vector profile demonstrates the induced circulating electric field around the nanobar, Fig. 3f–g.

**Fig. 4:**
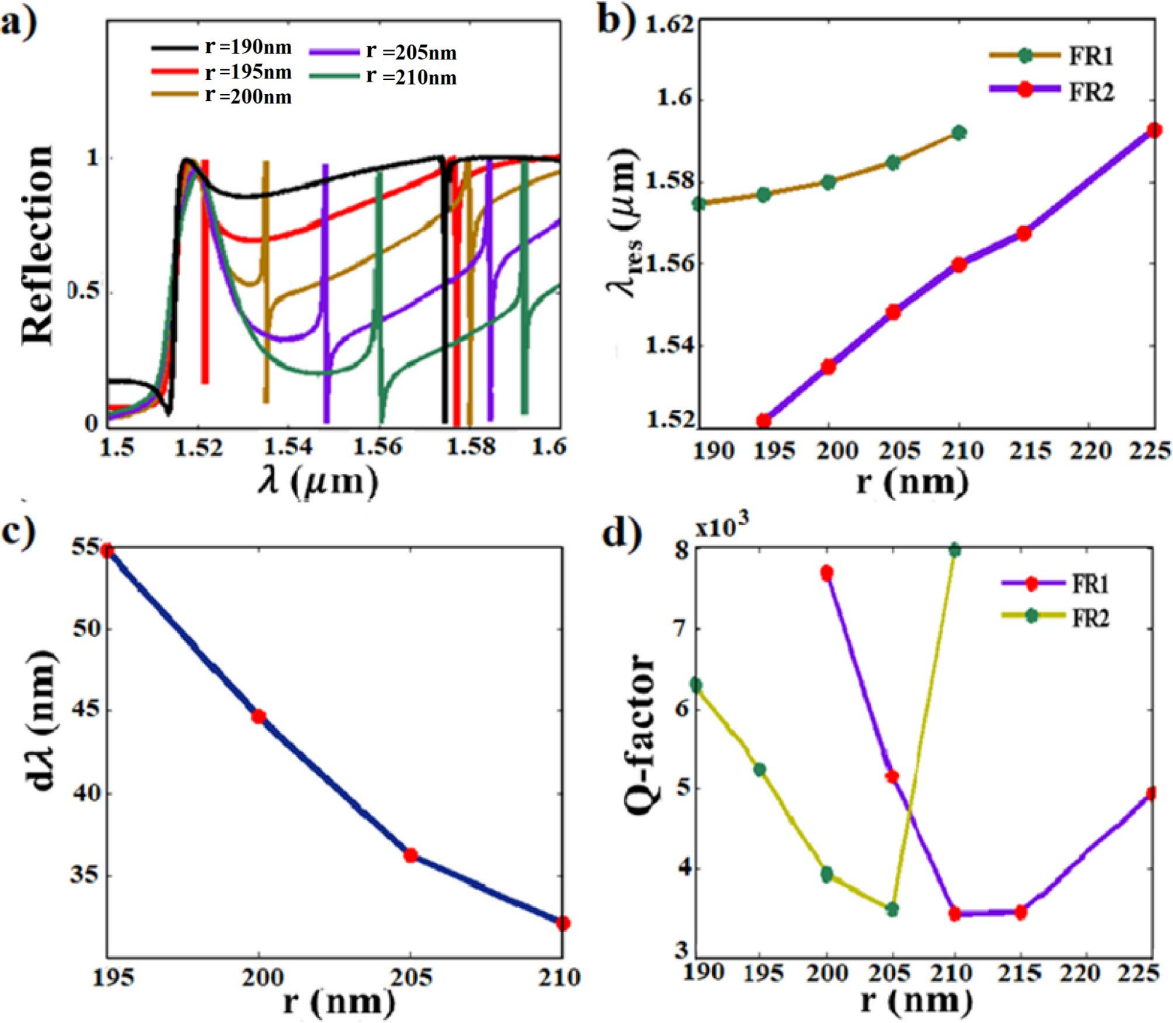
Effect of the radius parameter (r) on; (**a**) the normalized reflection spectrum, (**b**) the resonance wavelengths at FR1 and FR2, (**c**) the spectral separation between FR1 and FR2, and (**d**) the Q-factors of FR1 and FR2.

## The geometrical parameters’ effect on the double Fano resonances

Now, we show the effect of the geometrical parameters of the structure; the radius of the nanodisk, the gap distance between both resonators and the width of the nanobar resonator, in terms of the reflection spectrum, the resonance wavelengths, the spectral difference between the two resonances and the quality factor (Q-factor). Leveraging the Fano resonance mechanism explained in Sect. 6, we will explore how the geometrical parameter variations affect the design’s optical properties, providing the ability to optimize the design at will.

### The radius parameter of the nanodisk effect

First, we study the effect of varying the radius of the nanodisk (r), while keeping the gap distance fixed at $(G=130 nm)$ and the nanobar width fixed at (w = 300 nm). Figure 4a shows the reflection behavior of various nanodisk radius parameters (r) ranging from (r = 190 nm) to (r = 210 nm). The structure of (r = 190 nm) shows the emergence of Fano resonance at the spectral region of (FR2) only. By increasing the radius to (r = 195 nm), a transparency window is realized with a high dispersive nature that is considered an obstacle for sensing applications. Further increasing of the nanodisk radius shows the formation of significant double Fano resonances at FR1 and FR2. Increasing the radius (r) of the nanodisk from (r = 195 nm) to (r = 210 nm) causes a substantial red shift in the first Fano resonance (FR1) and a minor red shift in the second Fano resonance (FR2) as shown in Fig. 4b.

Hence, the spectral gap distances between the two resonances (dλ) decrease as demonstrated in Fig. 4c. The radius effect emphasizes that the FR1 is primarily attributed to the excited dark mode inside the nanodisk as previously explained. Further radius increasing than (r = 210 nm) diminishes the resonance at FR2. Figure 4d illustrates the calculated Q-factors for various radius parameters, revealing a maximum value of 7,674 for r = 200 nm at FR1 and 7,960 for r = 210 nm at FR2.

Aiming to operate within the telecommunication wavelength (λ = 1.55 μm), we select the structure with a radius of (r = 205 nm) that exhibits an operating wavelength of (λ = 1.548 μm) and Q-factor of 5,160 at FR1 for further investigations.

### The gap distance parameter effect

**Fig. 5:**
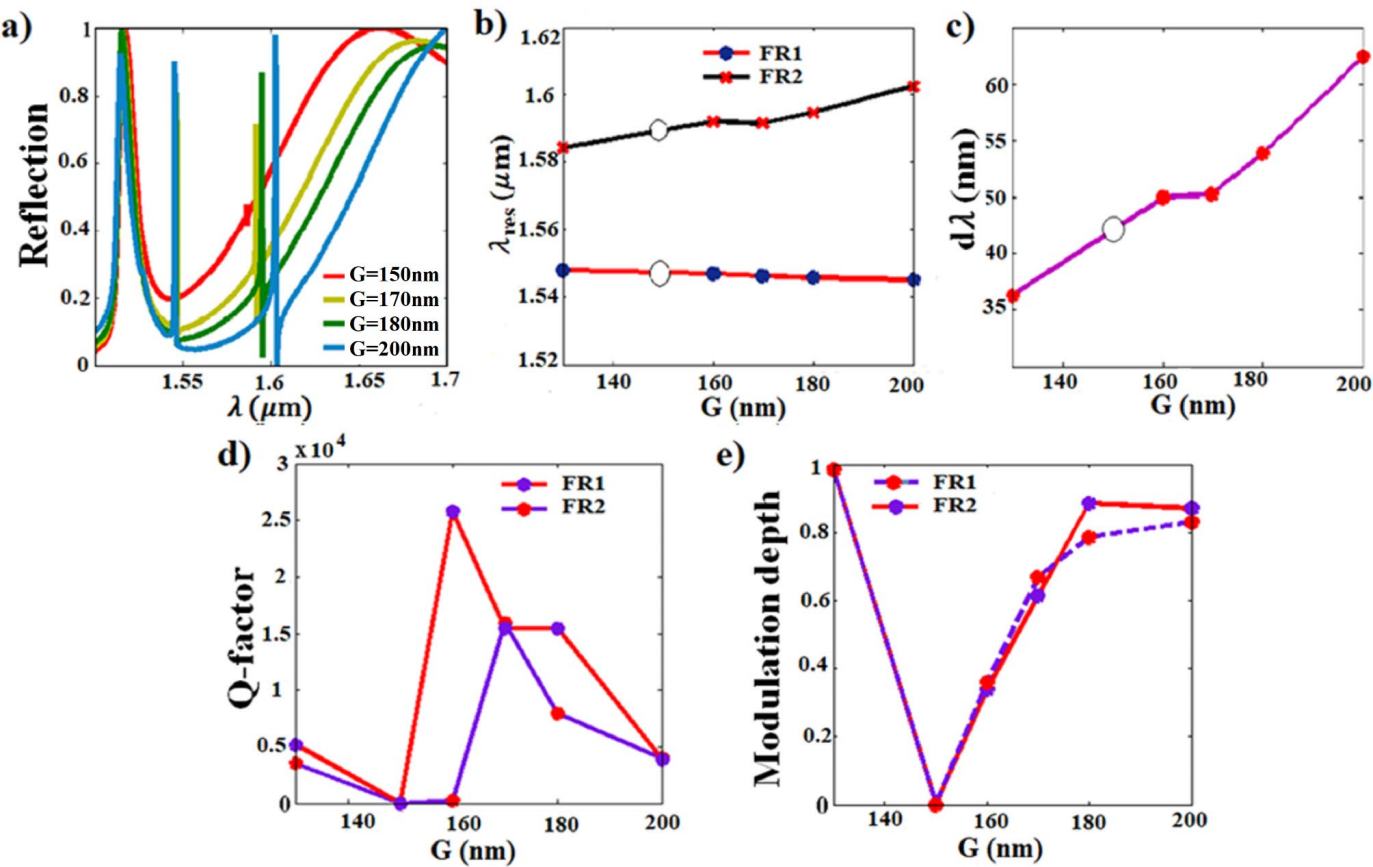
Effect of the (G) parameter on; (**a**) the normalized reflection characteristics, (**b**) the resonance wavelengths of FR1 and FR2 along the range of (G = 130–200 nm), (**c**) the spectral separation (dλ) between FR1 and FR2 (the open circle denotes the disappearing Fano resonance), (**d**) the calculated Q-factor, and (**e**) the modulation depth.

Next, we investigate the influence of varying one gap distance (G) between the nanodisk and the nanobar resonators, while keeping the other gap fixed at (G2 = 150 nm) on the reflection behavior as shown in Fig. 5. Increasing the G distance leads to opposing shifts: blue shift for FR1 and redshift for FR2 as depicted in Fig. 5a and Fig. 5b. Consequently, the spectral gap distance (dλ) between FR1 and FR2 widens with increasing G as illustrated in Fig. 5c. For G = 150 nm, the Fano resonance at both FR1 and FR2 disappears due to symmetry as may be observed from Fig. 5a (red curve). In addition, G exerts a substantial influence on the modulation depth that is enhanced as G increases beyond 150 nm showing a notable enhancement up to (~ 90%) for (G = 180 nm) as depicted in Fig. 5a and e. Additionally, the calculated Q-factors are noteworthy for G values between 160 and 180 nm, with FR1 exhibiting exceptional Q-factors of 25,784 for (G = 160 nm) and 15,459 for (G = 180 nm). However, the structure of (G = 160 nm) suffers from the limitation of the small modulation depth of 35% as may be observed in Fig. 5e. Therefore, the structure of (G = 180 nm) is the more suitable choice for further investigation.

### The nanobar width parameter effect

**Fig. 6:**
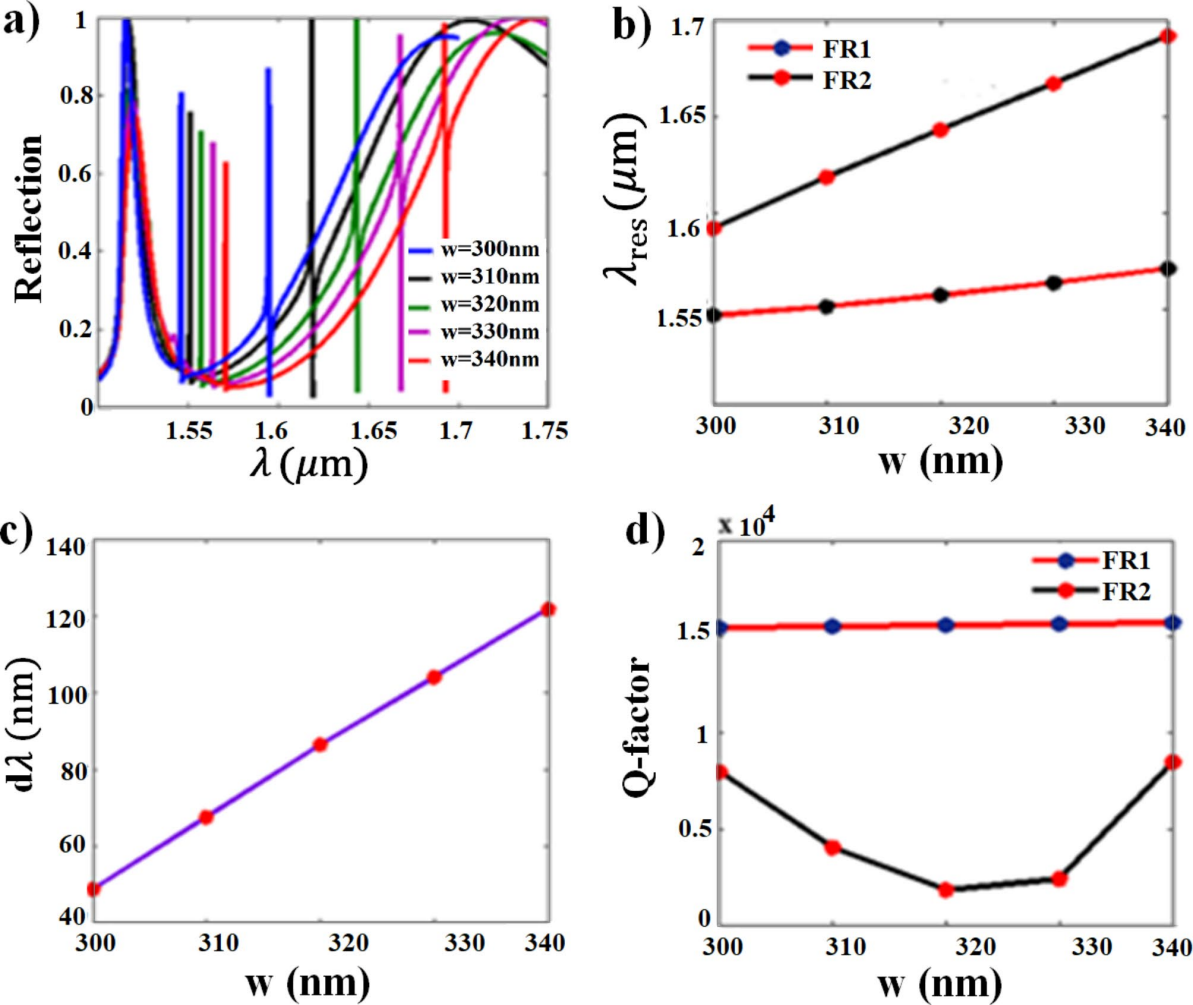
Effect of nanobar parameter width (w) on; (**a**) The normalized reflection spectra, (**b**) The resonance wavelengths, (**c**) The spectral separation between FR1 and FR2, and (**d**) The Q-factor of both resonances.

Further investigation is performed for the structure of different nanobar resonator widths. In contrast to increasing the nanodisk radius, increasing the nanobar width causes a substantial redshift to FR2 and a minor redshift to FR1, as can be observed from Fig. 6a and b. Increasing the nanobar width provides the advantage of increasing the spectral gap (dλ) between FR1 and FR2 as illustrated in Fig. 6c. The calculated Q-factors of FR1 retain at high values reaching up to 15,711 for the structure of (w = 340 nm), while the Q-factors of FR2 varies having its maximum 3,463 (in back scattering) for the structure of (w = 340 nm) as shown in Fig. 6d.

Having examined the individual effects of varying the nanodisk radius, gap distance, and nanobar width (as shown above), it becomes clear that these geometrical parameters significantly influence the Fano resonances (FR1 and FR2) in our design, supporting the mechanisms explained in Sect. 6. This influence arises from the confinement of electric and magnetic fields within the resonators. Elaborating on this point, since the resonating electric and magnetic fields are highly confined inside the nanodisk at FR1, any variation in the disk parameter significantly affects FR1 shift. The minor effect on FR2 results from the interference between the excited dark mode inside the disk with the bright mode of the nanobar. On the contrary, the variation of the nanobar width highly influences FR2, as the emergence of FR2 is attributed to the highly confined magnetic and electric field inside the nanobar. The lower Q-factor of FR2 as compared to FR1 is due to the leakage of the electric field to the gap distances on both sides of the nanobar resonator. The gap distance variation highly influences the Q-factor and the modulation depth, as shown in Fig. 5d and e. For the symmetrical gap distances on both sides of both resonators, the induced magnetic field in the dark mode is 180° out of phase of the bright mode resonator, hence, the fields destructively interfere and k (coupling coefficient) goes to zero, resulting in diminishing of the Fano resonance. Increasing one of the gap distances leads to constructive and destructive interference between the dark and bright modes, resulting in the Fano spectral line. Optimizing the gap distance is aiming at minimizing k, achieved at G = 160 nm, and consequently high confinement of the field and hence, a high Q-factor. The phase difference and k, affected by the gap distance, exhibit a significant impact on the Q-factor and modulation depth.

## Double Fano resonance application in gas sensing

Many gas molecules possess unique absorption fingerprints within the telecommunication wavelength range. For selective gas sensing applications, we excite the specific Fano resonance at a wavelength precisely matching the gas’s spectral fingerprint. This optimal alignment induces a remarkable spectral shift accompanied by significant losses due to absorption, highlighting the gas molecules’ unique spectral signatures.

**Fig. 7:**
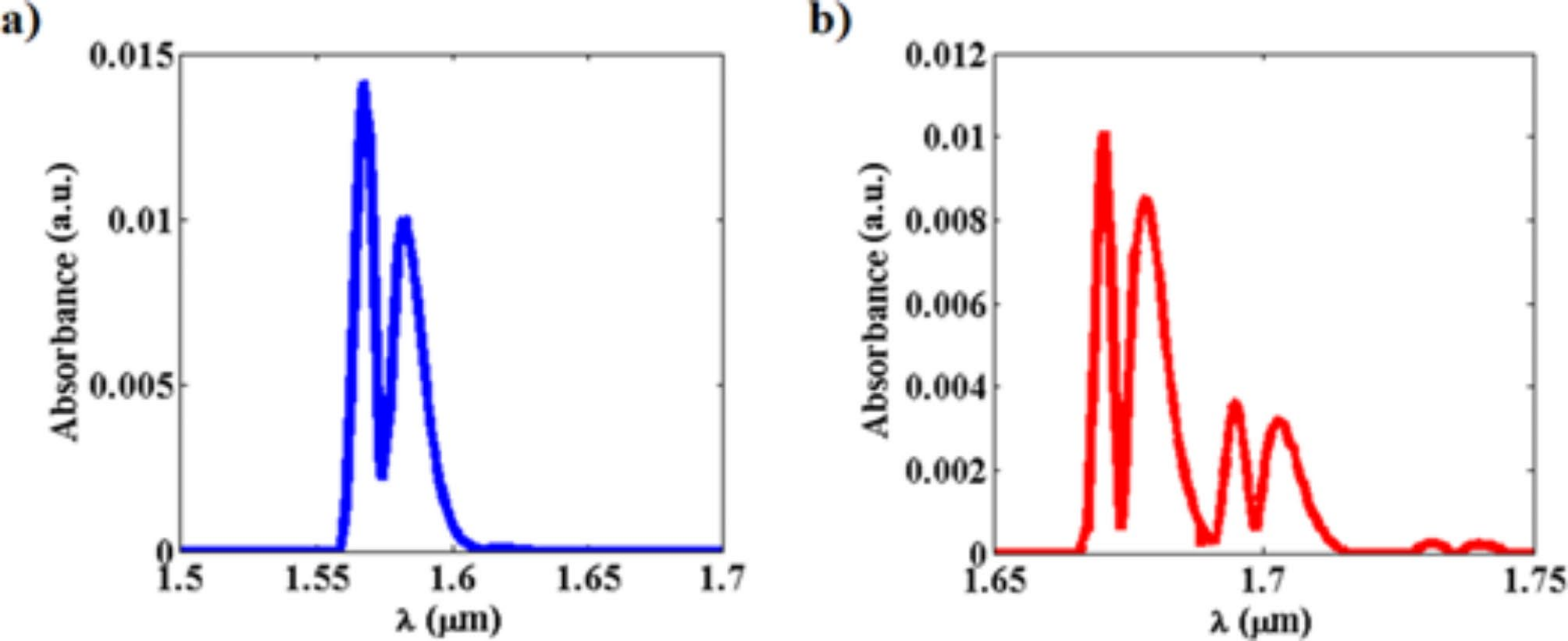
The absorbance of (**a**) Carbon monoxide (CO) and (**b**) Nitrous oxide (N_2_O) as extracted from the spectraplot simulation tool based on the HITRAN database^54,55^.

CO and N_2_O gases are significant greenhouse gases that contribute to climate change and environmental degradation^8,56^. The accurate and efficient sensing of these gases is crucial for mitigating their adverse impacts. CO and N_2_O possess distinct absorption fingerprints in the telecommunication wavelength range at (λ = 1.57 μm) and (λ = 1.67 μm), respectively. The absorbance of both gases at temperature (T = 298° K), pressure (P = 1 atm), effective path length (l = 5m) and gas mole-fraction (X = 0.01) are computed using Spectraplot tool based on HITRAN database and presented in Fig. 7^54,55^. Using the absorbance data, the dispersive real and the imaginary parts of the complex refractive index of CO and N_2_O are calculated using the Krammers-Kronig relation^57^:

10 $$n\left( {{{\uplambda }}_{0} } \right) = ~n\left( {{{\uplambda }}_{1} } \right) + p\frac{{\left( {{{\uplambda }}_{1}^{2} - {{\uplambda }}_{0}^{2} } \right)}}{\pi }~\mathop \int \limits_{0}^{\infty } \frac{{{{\uplambda k}}\left( {{\uplambda }} \right){{d\uplambda }}}}{{\left( {{{\uplambda }}_{1}^{2} - {{\uplambda }}^{2} } \right)\left( {{{\uplambda }}_{1}^{2} - {{\uplambda }}^{2} } \right)}}$$

where $p$ is the Cauchy principal value of integral, $n\left({\uplambda }_{1}\right)$ is the known refractive index of the gas at wavelength (${\uplambda }_{1}$) and $\text{k}(\uplambda )$ extinction coefficient that is calculated from the absorption coefficient ${\upalpha }(\uplambda )$ as follows:

11 $${\text{k}}\left( {\uplambda } \right) = \frac{{4\pi {\upalpha }\left( {\uplambda } \right)}}{\lambda }$$

**Fig. 8:**
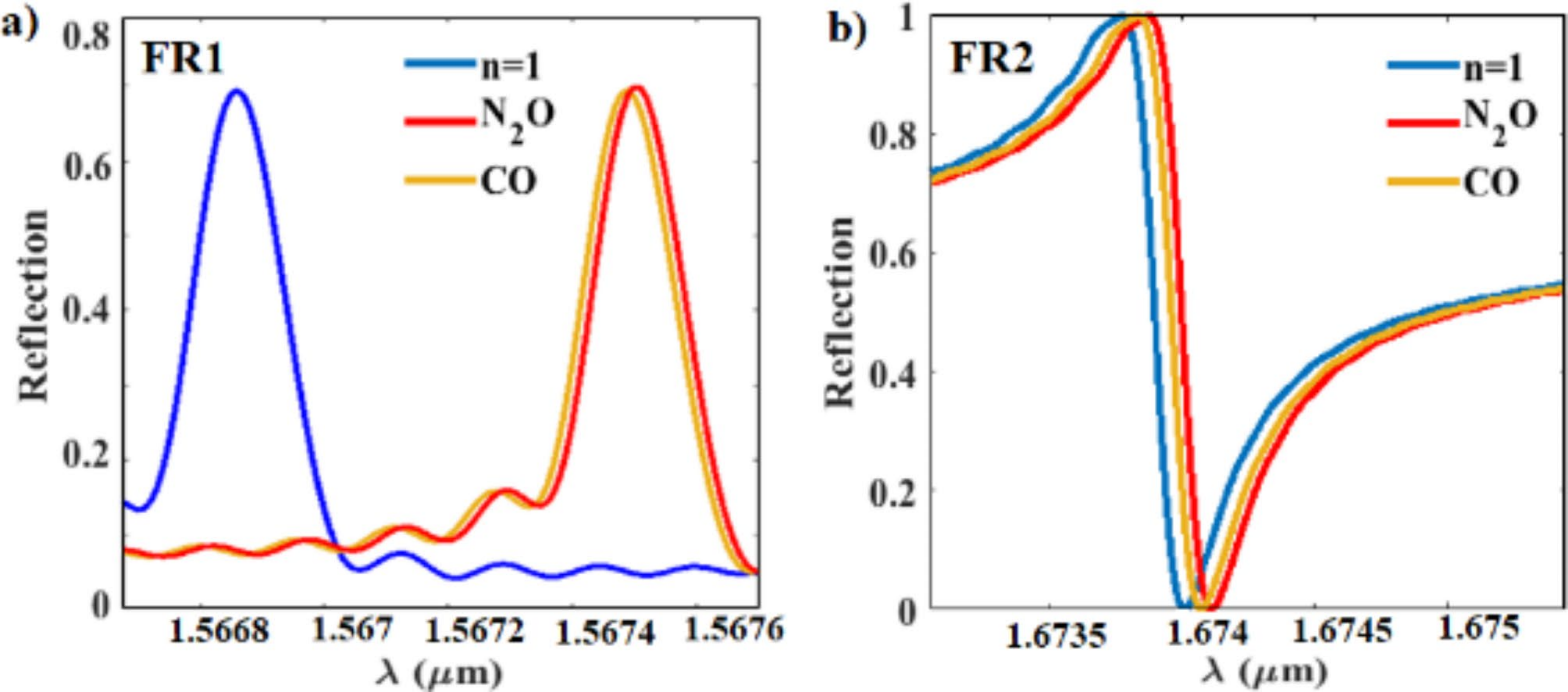
The normalized reflection spectra of the gas sensor with a vacuum (n = 1) as a reference towards CO and N_2_O at; (**a**) FR1 and (**b**) FR2.

Leveraging the unique optical properties of double Fano resonances, we have developed our design for sensing both CO and N_2_O gases. The structure is optimized with parameters; r = 205 nm, G = 180 nm, and w = 333 nm, enabling the realization of double Fano resonance at λ = 1.5667 μm and λ = 1.674 μm. The normalized reflection spectra of the sensor for both CO and N_2_O with respect to the vacuum (n = 1) at both resonances are presented in Fig. 8. Our sensor design exhibits exceptional sensitivity at FR1 to CO (S_1CO_ = 1,735 nm/RIU) and less sensitivity to N_2_O (S_1N2O_ = 1,240 nm/RIU). However, a larger spectral shift for N_2_O is observed due to its higher refractive index (${n}_{r}\approx$ 1.000516) compared to CO (${n}_{r}\approx$ 1.000363), as depicted in Fig. 8a. The higher sensitivity to CO than N_2_O is attributed to Eq. (2), which emphasizes that matching the resonant frequency of the sensor with the fraction of optical energy of the resonant mode (F) in the analyte, CO in this case, as well as decreasing the real part of the refractive index contribute to enhancing the sensitivity of the sensor. Similarly, the sensor exhibits higher sensitivity at FR2, which matches the resonant mode of N_2_O, to N_2_O (S_2N2O_ = 194 nm/RIU) than to CO (S_2CO_ = 165 nm/RIU), with higher spectral shift to N_2_O, see Fig. 8b.

**Fig. 9:**
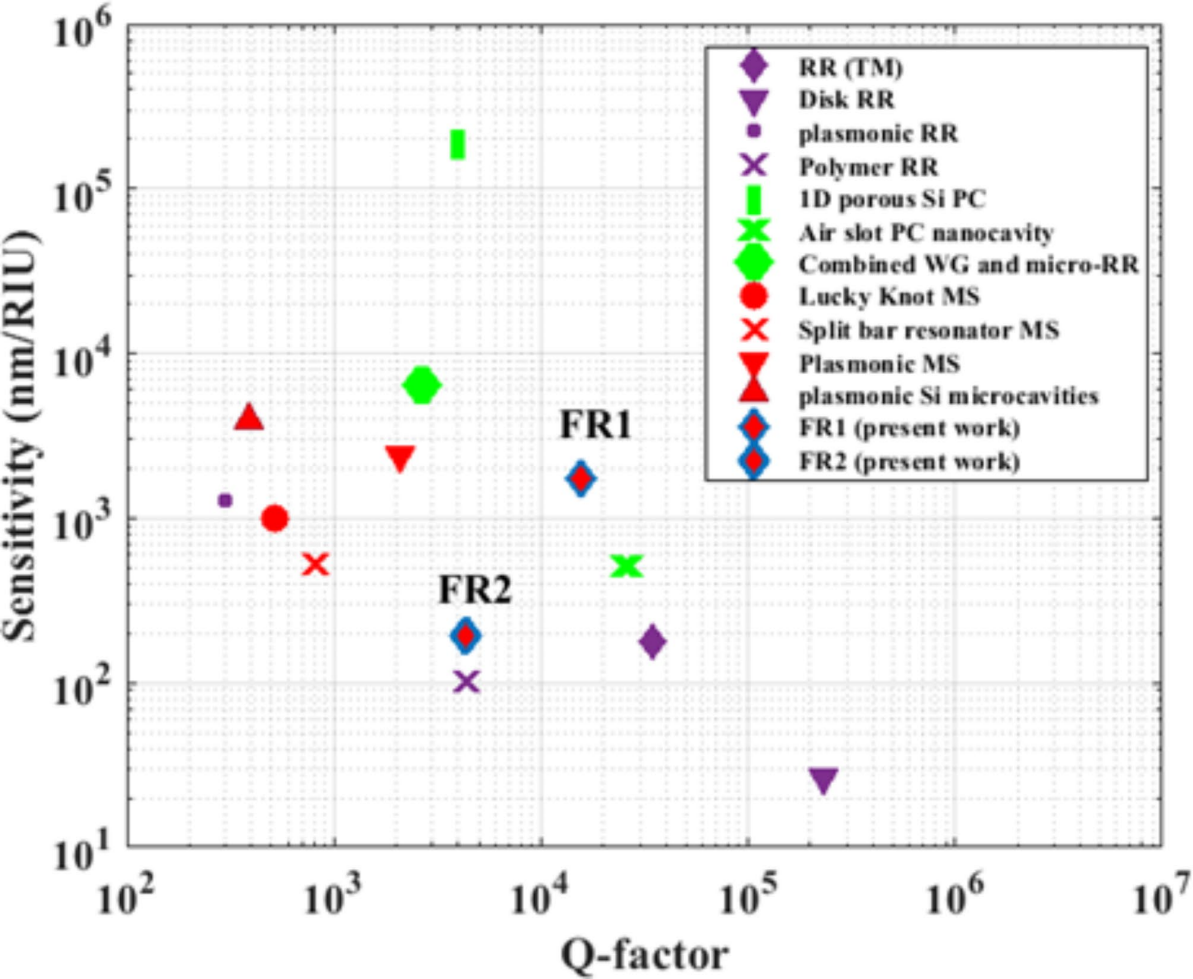
Summary of sensitivity performance of different platforms including:RR (purple markers), PC (green markers), metasurfaces MS (red markers), and the present metasurface sensor (the blue outlined red triangle).

Furthermore, the structure demonstrates a significant high Q-factor ($>{10}^{4}$) at FR1, which decreases by 6.3% due to the absorption of CO, while it remains with no change upon exposure to N_2_O, in agreement with Eq. (5). This decrease in Q-factor serves as a selectivity parameter of CO. In terms of FOM, the sensitivity of FR1 to CO (1.4 times sensitivity towards the N_2_O), along with the slight decrease in Q-factor lead to a FOM ratio (FOM_CO_/ FOM_N2O_ = 1.311), where FOM_CO_ is of (10,844) and FOM_N2O_ is of (8,266).

On the contrary, the Q-factor, calculated for the peak of FR2, doesn’t change upon exposure to both gases. The Q-factor, calculated at the dip, is decreased with the same amount for both N_2_O and CO by ~ 2.6%. This reveals that at FR2 the change in Q-factor is not useful to differentiate between the two gases and the selectivity is primarily dependent on the spectral shift due to the change of the real part of the index of refraction. This observation can be explained using Eq. (5), as $\frac{1}{{Q}_{tot}}$ at FR2 is controlled by the high value of the first term of $\left(\frac{1}{{Q}_{d}}\right)$, which results in negligible effect of the minor change in the second term $\left(\frac{1}{{Q}_{a}}\right)$. In terms of FOM, a FOM ratio (FOM_N2O_/ FOM_CO_ = 1.214), where FOM_N2O_ is of (510) and FOM_CO_ is of (420). The sensor’s sensitivity performance at FR2 is lower than its performance at FR1, but it is still good enough to be useful for many applications.

Noteworthy, the upper limit of FOM for CO and N_2_O at their absorption fingerprints can be calculated by Eq. (7), resulting in $1.4518\times {10}^{6}$ and $1973\times {10}^{6}$, respectively. Away from the fingerprints, ${FOM}_{max}$ tends towards infinity.

Our proposed sensor offers an excellent compromise between the sensitivity and Q-factor, offering a significant FOM as compared to other sensor configurations; ring resonator (RR), photonic crystal (PC), and other metasurface designs. In Fig. 9, we represent the Q-factor versus the sensitivity of different configurations of various platforms. FOM is directly proportional to sensitivity and Q-factor. LOD is inversely proportional to sensitivity and Q-factor. Hence, the data points of higher FOM and better LOD are found towards the upper-right of the plot. The configurations of (RR), denoted by purple marker, including Si RR^30^, polymer RR^58^, and disk RR^30^, demonstrate low sensitivities compared to our design. PC configurations are denoted by the green marker. A 1D porous Si PC integrated with an Ag layer deposited on a prism exhibits exotic sensitivity with high Q-factor^59^. However, the experimental realization of the deposited Ag layer on a dielectric is complicated^60^. PC based on combined waveguide (WG) and micro-RR demonstrates significant sensitivity with remarkable Q-factor, although the difficulty of fabrication of the periodic rods imposes great challenges^61^. Air slot PC nano-cavity also exhibits extremely high Q-factor^62^, although the periodic lattice of drilled holes, apart from the fabrication challenges, is expected to introduce surface roughness and further scattering losses^30^. Metasurface structures including; Lucky Knot^42^, split bar resonator^43^, and plasmonic metasurfaces^43^, in addition to plasmonic Si microcavities^37^ exhibit high sensitivities with low Q-factors. This comparison highlights the superiority of our design performance, which shows a significant enhancement of FOM (11,570) and LOD ( $8.6\times {10}^{-5}$) at FR1. In addition, the high Q-factor of FR1 offers promises for high selective sensors at the operating telecommunication wavelengths. Furthermore, a sensor at FR2 can serve as a good selective gas sensor based on the sensitivity to the minor change of the real part of the refractive index. Moreover, our design provides flexibility in engineering Fano resonances through structure optimization of different geometrical parameters.

## Conclusion

This study reports the development of a selective gas sensor based on double Fano resonance operating at telecommunication wavelengths for PIC. The sensor design employs an all-dielectric silicon metasurface consisting of coupled nanodisk and nanobar resonators. Each Fano resonance can be engineered independently by adjusting the geometrical parameters, including the nanodisk radius, the gap between the nanodisk and the nanobar, and the nanobar width. Leveraging Fano resonance at the absorption band of the sensing material holds promise for selective gas sensing applications. We demonstrate the feasibility of this approach by developing a sensor design capable of sensing both CO and N_2_O gases at wavelengths of 1.566 µm and 1.674 µm, respectively. The sensor exhibits remarkable sensitivities of 1,750 nm/RIU for CO and 194 nm/RIU for N_2_O, accompanied by exceptional FOM of 11,570 and 510, respectively. The absorption losses, evident from the decreased Q-factor, are identified revealing values of 6.3% for CO at FR1. The low Q-factor of FR2 limits the selectivity to the spectral shift, while the absorption losses are 2.6% for N_2_O and CO, as well. These distinct absorption losses accompanied by the spectral shift highlight the proposed design’s potential as a highly sensitive and selective gas sensor. Additionally, the sensor’s compatibility with CMOS technology and its low-cost fabrication process make it an attractive candidate for practical applications.

## Materials and methods

Finite difference time domain (lumerical software) has been used for simulating the optical response of the proposed structures to incident plane waves. Matlab software has been used for calculating Krammers Kronig’s relation.

## Electronic supplementary material

Below is the link to the electronic supplementary material.

Supplementary Material 1

## Data Availability

The datasets used and/or analysed during the current study available from the corresponding author on reasonable request.
