# Supplementary material for: Silicon-based double fano resonances photonic integrated gas sensor
Source: Sci Rep. 2024 Oct 22;14:24811. doi: 10.1038/s41598-024-74288-6 (PMC11496707; doi:10.1038/s41598-024-74288-6)
Supplement: Supplementary file 1 — Supplementary Material 1 [file 41598_2024_74288_MOESM1_ESM.docx]

**Silicon-based Double Fano resonances photonic integrated gas sensor**

[Norhan A. Salama](https://www.nature.com/articles/s41598-020-61639-2#auth-Norhan_A_-Salama)^1, 2^, Shaimaa M. Alexeree^1^, [Salah S. A. Obayya](https://www.nature.com/articles/s41598-020-61639-2#auth-S__S__A_-Obayya)^3^, [Mohamed A. Swillam](https://www.nature.com/articles/s41598-020-61639-2#auth-Mohamed_A_-Swillam)^2*^

^1^Laser Applications in Metrology, Photochemistry and Agriculture, National Institute of Laser Enhanced Sciences, Cairo University, Giza, Egypt

^2^Department of Physics, School of Science and Engineering, The American University in Cairo, Cairo, 11835, Egypt

^3^Centre for Photonics and Smart Materials, Zewail City of Science, Technology and Innovation, Giza, Egypt

*Corresponding author: m.swillam@aucegypt.edu

**Validation of the simulation results**

The validation of our simulation has been presented in the supplementary material. The validation is based on a previous literature [1], that uses a coupled nanodisk and nanobar resonators of poly crystalline Si resonators with a refractive index approximated to (n$\approx3.72)$ mounted on a quartz substrate of (n=1.48). The geometrical parameters are as follows; nanodisk radius (r=210nm), the nano-bar width (w=200nm), the nano-bar length (L = 700 nm), the first gap distance (G = 60 nm), the pitch in x-direction (p1 = 750 nm), the pitch in y-direction (p2 = 750 nm) and the thickness (t = 120 nm). Figure S1 presents the comparison between the transmission spectra obtained from our simulations and the reference. As can be seen from the figure, the resonance wavelengths and overall spectral features exhibit good agreement between our simulations and the reference data.

| **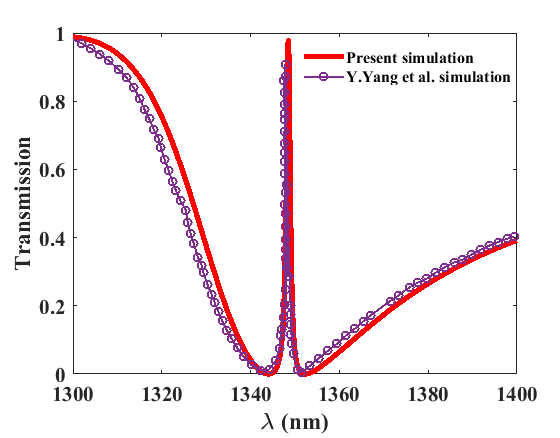** |
| --- |

Figure S1: Comparison of transmission spectra of the present work simulation (red line) and the Y.Yang et al. simulation (purple dotted line) [1]**.**

**References**

[1] Y. Yang *et al.*, “Nonlinear Fano-Resonant Dielectric Metasurfaces,” *Nano Lett.*, vol. 15, no. 11, pp. 7388–7393, 2015, doi: 10.1021/acs.nanolett.5b02802.
